# Bilateral Tubal Ectopic Pregnancy Following Clomiphene Administration: A Case Report

**DOI:** 10.7759/cureus.28977

**Published:** 2022-09-09

**Authors:** Alicia M Benz, Caitlin C Price, Fernando J Ocon

**Affiliations:** 1 Obstetrics and Gynecology, Lincoln Memorial University DeBusk College of Osteopathic Medicine, Houston, USA; 2 Obstetrics and Gynecology, HCA Houston Healthcare Southeast, Pasadena, USA

**Keywords:** chlamydia, salpingostomy, clomiphene, salpingectomy, bilateral ectopic pregnancy

## Abstract

A bilateral tubal ectopic pregnancy is the rarest form of extrauterine pregnancy and is extremely difficult to diagnose due to an initial presentation that is nearly indistinguishable from unilateral tubal ectopic pregnancy. We report a case of a 27-year-old primigravida woman who presented to our clinic with mild cramping and vaginal bleeding following the administration of clomiphene. Ultrasound confirmed the presence of a left-sided ectopic pregnancy. The patient underwent laparoscopy and was found to have a mass in the contralateral fallopian tube. Histologic evaluation of the specimens from both fallopian tubes confirmed the presence of a simultaneous bilateral tubal ectopic pregnancy. While no current guidelines exist for the management of bilateral ectopic pregnancies, we stress the importance of direct visualization of both fallopian tubes during laparoscopy for any suspected ectopic pregnancy.

## Introduction

Bilateral tubal ectopic pregnancy (BTP) is the rarest form of extrauterine pregnancy, occurring in approximately 1 in 725 to 1 in 1580 of all ectopic pregnancies [1], which is equivalent to 1 in every 200,000 live births [2]. BTP is initially difficult to diagnose, as its clinical presentation is nearly identical to unilateral tubal ectopic pregnancy. Rarely, it is diagnosed before surgery [3]. Furthermore, it is suspected that the incidence of BTP is underdiagnosed and is growing as assisted reproductive technology (ART) becomes more common [1]. Although the greatest risk factor for an ectopic pregnancy is a history of a previous ectopic pregnancy, pelvic infection (eg, chlamydia, gonorrhea, or nonspecific salpingitis) and a history of pelvic inflammatory disease (PID) are also major causes of tubal pathology, which ultimately increases the risk of ectopic pregnancy [3]. Other risk factors for tubal pregnancies include previous tubal surgery, intrauterine device use, in vitro fertilization, oral estrogen/progestin oral contraceptive usage, and smoking [4]. Furthermore, prolonged clomiphene use is also associated with an increased risk of ectopic pregnancy [5].

Clomiphene, a nonsteroidal triphenylethylene derivative, acts as a selective estrogen receptor modulator (SERM) in the hypothalamus and inhibits the negative feedback of circulating endogenous estradiol [4]. As a result, hypothalamic gonadotropin-releasing hormone (GnRH) pulse frequency is increased, increasing follicle-stimulating hormone (FSH) and luteinizing hormone (LH) concentrations [4]. Clomiphene is commonly used to induce ovulation in women desiring pregnancy and frequently can cause poly-ovulation, which may increase the risk of a BTP [1].

Although the exact mechanism of BTP is unknown, it is postulated to be due to the transperitoneal migration of trophoblastic cells [6]. Other theories include multiple ovulations and the oocyte implanting on a site of tubal damage [7] or superfetation - fertilization of an ovum in an already pregnant female [6]. It is hypothesized that clomiphene induces apoptosis of epithelial cells in the isthmus, slowing down embryo passage in the fallopian tube, which results in an ectopic pregnancy [5]. However, there is little, if any, research regarding the association between clomiphene use and bilateral ectopic pregnancy [1]. This is likely due to both the rarity and the under-reported incidence of BTP [1].

Here, we report a case of a 27-year-old woman with a BTP following clomiphene use. The patient was found to have evidence of chronic salpingitis during laparoscopy. This case adds to the medical literature by describing a previously unreported BTP with a history of both clomiphene use and chlamydia infection.

## Case presentation

A 27-year-old primigravida woman with a past medical history of polycystic ovarian syndrome (PCOS) and previously treated chlamydial infection was seen by our advanced practice provider for infertility secondary to PCOS. She was started on an initial trial of 50 mg clomiphene daily for five days. She presented to our clinic nine days after she reported a positive at-home pregnancy test for intermittent vaginal bleeding. Her estimated gestational age at this appointment was 5 weeks and 4 days, based on her last menstrual period. Transvaginal ultrasound (TVUS) showed an empty uterus with an endometrial stripe of 1.31 cm (normal: 0.4 cm). Serum beta-human chorionic gonadotropin (hCG) returned at 4,988 mIU/mL. Given her known Rh-negative blood type, 1500 units of intramuscular RhoGam were administered as the diagnosis of threatened abortion was made. She was instructed to have her beta-hCG repeated two days later; it returned elevated at 8,712 mIU/mL.

**Figure 1:**
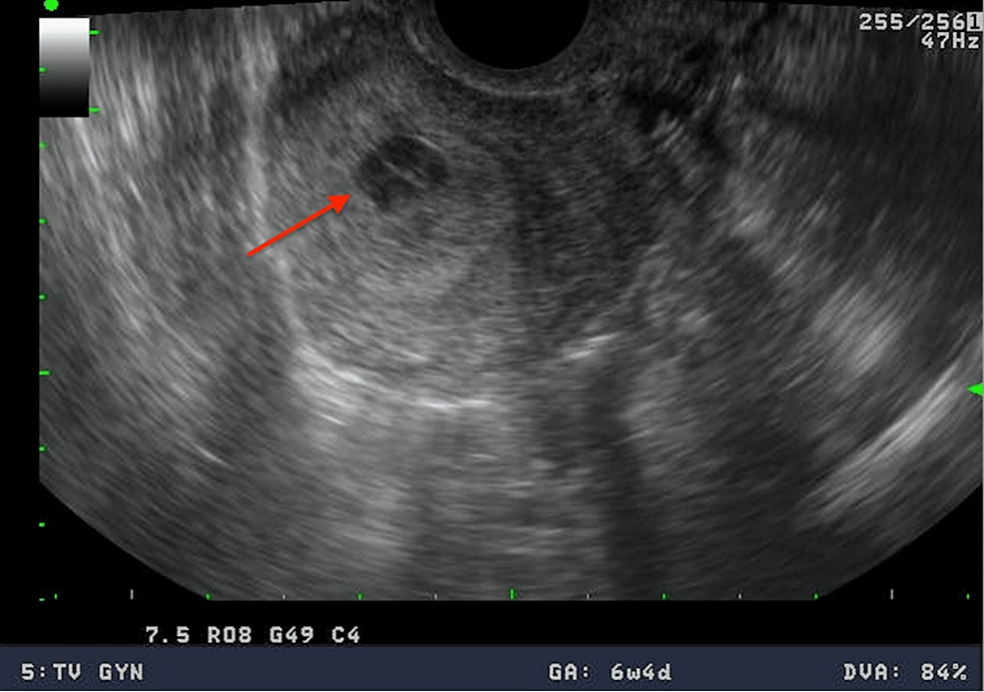
Transvaginal ultrasound demonstrating a possible intrauterine gestational sac following clomiphene administration

The patient was seen in the clinic five days following this second beta-hCG lab draw for vaginal spotting and cramping. A TVUS was repeated and showed a possible intrauterine gestational sac with debris consistent with blood clots as shown in Figure 1. The diagnosis of incomplete abortion was made. She was offered surgical versus conservative treatment and opted for medical management. She was given 3 tablets of 200 mcg misoprostol to administer intravaginally every 8 hours.

**Figure 2:**
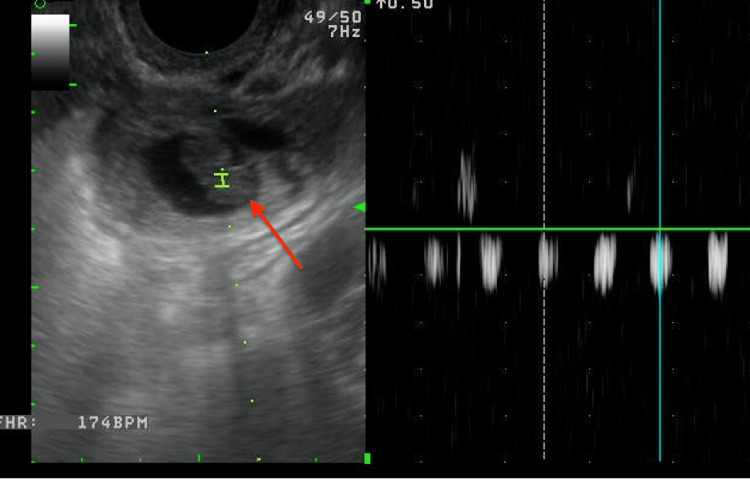
Left adnexal embryo with positive fetal heart tones

The patient successfully completed her misoprostol and presented two weeks later for a follow-up to confirm there were no products of conception in the uterus. She did note that she had continued to experience pelvic discomfort and vaginal bleeding with clots. Repeat TVUS showed an empty uterus and a thickened endometrium >2 cm. A tubal pregnancy with a fetal heart rate of 174 BPM was identified in the left adnexa, as shown in Figure 2. The beta-hCG level at this time was 34,473 mIU/mL. Due to the presence of fetal heart tones and elevated hCG, a diagnostic laparoscopy was immediately pursued.

**Figure 3:**
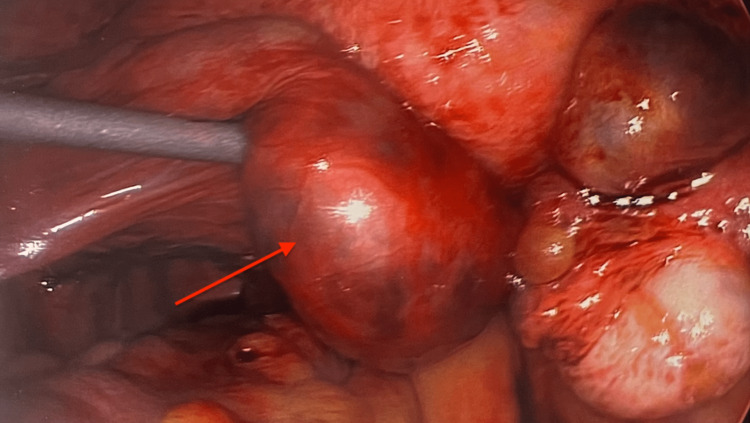
Left ectopic pregnancy with evidence of chronic inflammation

**Figure 4:**
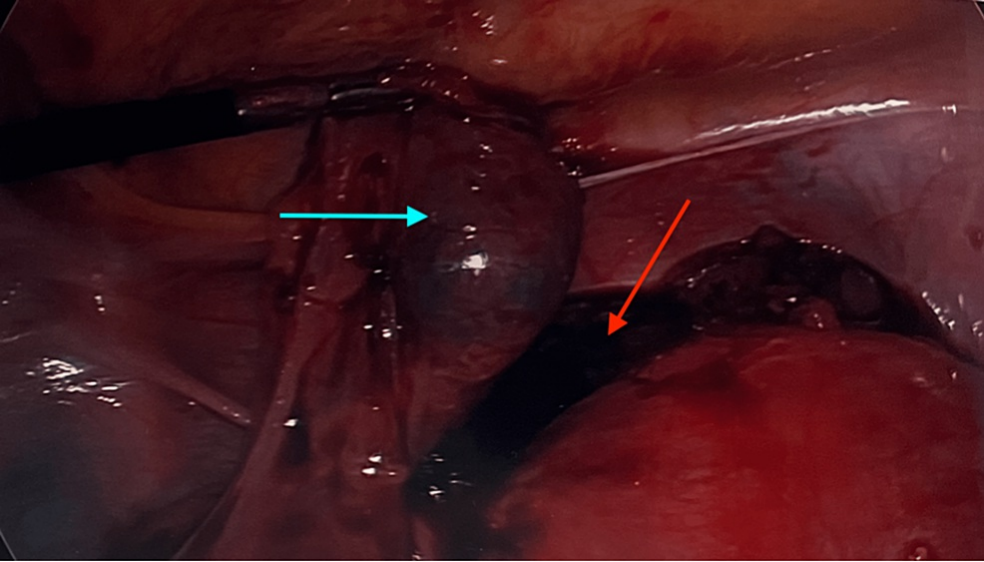
Right tubal mass (blue arrow); blood in the cul-de-sac (red arrow)

Laparoscopic findings showed chronic salpingitis bilaterally, with oophoritis on the left, as shown in Figure 3. Hemoperitoneum was noted, as well as bleeding from the left fallopian tube, which appeared to be damaged. A left salpingostomy was performed and the ectopic pregnancy was evacuated. After the salpingostomy was completed, the left fallopian tube was investigated further and, unfortunately, showed no viable fimbriae or viable tissue to support future conception or pregnancy. Thus, it was decided to proceed with salpingotomy. Upon visualization of the right fallopian tube, there appeared to be a large, apparent hemorrhagic mass consistent, but not definitive with, another ectopic pregnancy, as shown in Figure 4. Thus, a salpingectomy was also performed on the patient’s right side. Due to the presence of a possible gestational sac on this patient’s previous TVUS, dilation and curettage were performed with endometrial tissue collected. The patient tolerated the procedure well, and there were no intra-operative complications.

The specimens from both the left and right fallopian tube masses were sent to pathology in separate containers to avoid contamination with fetal cells where the presence of a simultaneous BTP was confirmed. The biopsy of the endometrium was also sent to pathology individually where no hydrophilic villi or discrete embryonic or fetal tissue was seen, thus confirming the absence of an intrauterine gestation.

Since bilateral salpingectomies were performed, serial beta-hCG levels were not needed postoperatively. The patient recovered well without any postoperative complications. In vitro fertilization (IVF) was discussed with the patient for future efforts of conception.

## Discussion

BTP is rare, occurring in approximately 1 per 200,000 pregnancies [4]. Although BTP has been documented since the 1940s, there has been an increase in the incidence of BTP secondary to the advancements in assisted reproductive techniques [8]. Clomiphene, a selective estrogen receptor modulator, has been widely used to induce ovulation for the past 40 years. A study completed in 1989 by Dickey et al. suggested that there was no difference in the risk of unilateral or bilateral ectopic pregnancies in patients receiving clomiphene for ovulation induction in patients without a history of pelvic inflammatory disease [9]. Unfortunately, there have not been any recent studies completed on the matter.

Based on our literature review, the first reported case of a simultaneous BTP following clomiphene use was in 1977 [10]. Many of the most recent studies do not report a history of pelvic inflammatory disease or sexually transmitted infection (STI). This may be due to the fact that more recent reports of BTP did not include chlamydial antibody titers [1].

The mechanism of BTP is postulated to be due to the transperitoneal migration of trophoblastic cells [6]. Other theories include multiple ovulations and the oocyte implanting in a site of tubal damage [7] or superfetation - fertilization of an ovum in an already pregnant female [6]. Our patient had a history of chlamydial infection, which was treated and confirmed with a test of cure. Given that pelvic inflammatory disease (PID) increases the risk of a unilateral ectopic pregnancy, it is our hypothesis this patient’s history of PID increased her risk of BTP. This, in combination with her clomiphene ovulation induction, may have resulted in multiple ovulations and thus, BTP.

A meta-analysis completed by Xia et al. looked at 25 studies with 11,960 patients and showed that there was a significant relationship between chlamydial infection and unilateral ectopic pregnancy (OR, 3.03; CI 95%, 2.37-3.89) [11]. While a history of STI is a well-established risk factor for developing a unilateral ectopic pregnancy, there is no case report of a bilateral ectopic pregnancy plus a history of an STI. This calls for further research into the association between the history of STIs such as chlamydia and the risk of developing a BTP, as chlamydia is the most common STI in the United States, according to the CDC's STI Prevalence, Incidence, and Cost Estimates.

Unfortunately, the clinical symptoms or serum beta-hCG cannot differentiate a unilateral from bilateral ectopic pregnancy, thus, the diagnosis of BTP is typically concluded at the time of surgery, as it is extremely difficult to identify abnormalities of the contralateral tube on ultrasound [1]. This is in part due to the rareness of the condition or obscured visibility from the presence of blood in the pelvis [1]. One literature review completed by Ramadan et al. found that only 7/45 (15.5%) of BTP were diagnosed preoperatively with transvaginal ultrasound [1]. 

Management depends on the extent of the damage to the fallopian tube and the desirability of future fertility. According to the American College of Obstetricians and Gynecologists (ACOG) practice bulletin No. 193, methotrexate is the treatment of choice for the medical management of ectopic pregnancy in those who do not have absolute contraindications. Instead, these patients are typical candidates for surgical intervention. Our patient’s elevated beta-hCG of >5000 and the presence of fetal heart tones are known relative contraindications to methotrexate administration [12]. Therefore, surgical intervention was pursued.

Regarding treatment, there are no guidelines available on the management of BTP. Ultrasound typically diagnoses an ectopic pregnancy in one tube and the contralateral ectopic is found during laparoscopy. The decision to perform a salpingostomy versus salpingectomy is determined by the patient’s desire for future fertility and the extent of fallopian tube damage [12]. This emphasizes the importance of maintaining a high level of suspicion at the time of surgical intervention and of visualizing the contralateral tube during laparoscopy to avoid missing a potential second ectopic tubal pregnancy [13]. One cohort study found that salpingostomy is associated with higher rates of subsequent intrauterine pregnancy but also a higher risk of a repeat ectopic pregnancy [14]. When significant fallopian tube damage is visualized, salpingectomy is the preferred method of treatment, as seen in our patient.

**Figure 5:**
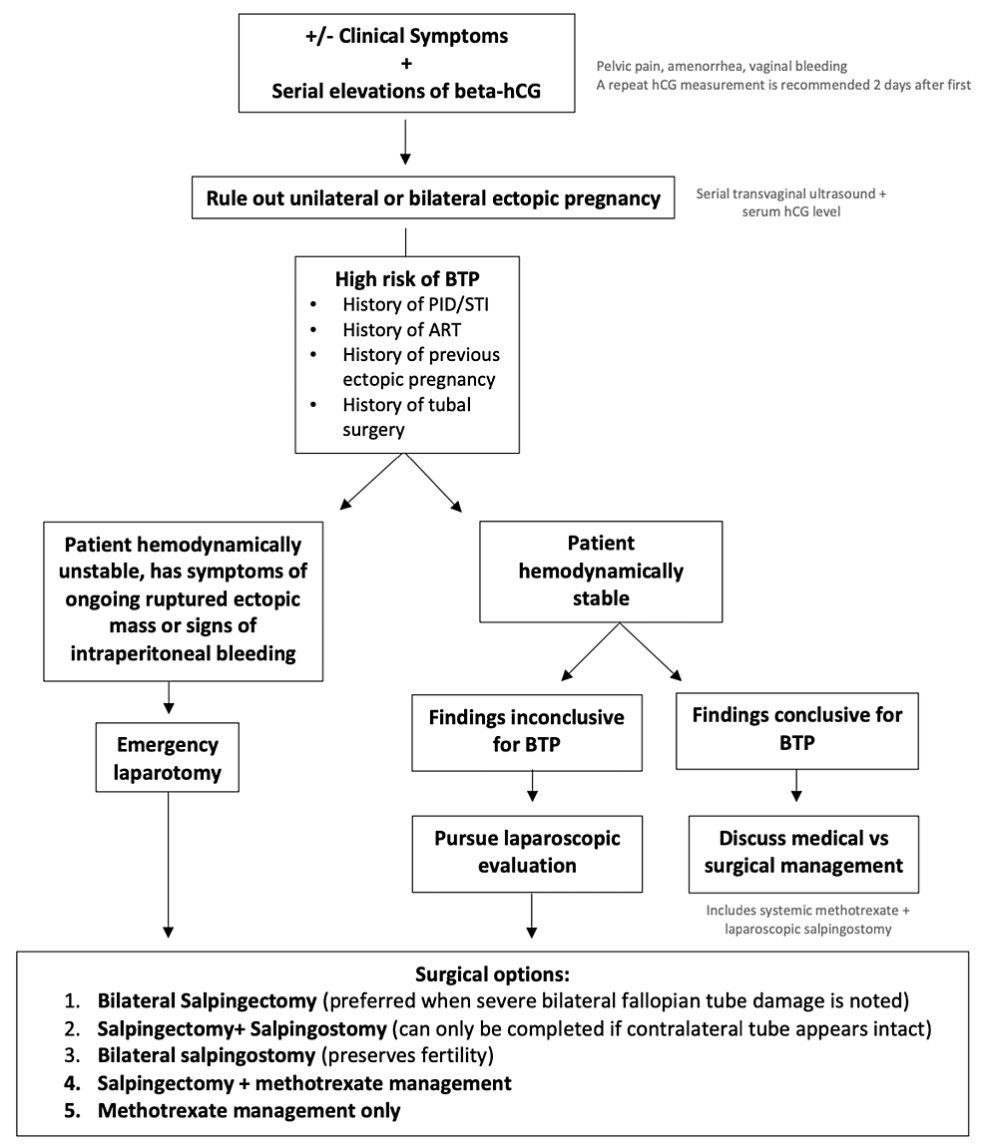
Algorithm for management of BTP BTP = bilateral tubal ectopic pregnancy; PID = pelvic inflammatory disease; STI = sexually transmitted infection; ART = artificial reproductive technology

As there is minimal information with regard to the management of BTP, we propose a simple algorithm (Figure 5) for patients at risk of BTP to minimize the possibility of missing or misdiagnosing a BTP. This method also discusses potential management options. Information from Figure 5 is supported by ACOG Practice Bulletin No. 193 and Ghosh et al. [12,15].

## Conclusions

We report this case to demonstrate the need for a careful assessment of both fallopian tubes laparoscopically in a suspected unilateral ectopic pregnancy, as a BTP is commonly missed on ultrasound preoperatively. As the use of clomiphene is becoming increasingly common, the incidence of BTP may rise as well. We encourage providers to discuss the possibility of a bilateral ectopic pregnancy with their patients who are at a higher risk, including those who have undergone recent clomiphene use or other artificial reproductive technology, especially in those with a history of sexually transmitted infection. Given that there are no current guidelines regarding the management of BTP, further research is needed to investigate all potential diagnostic and treatment modalities.
